# Molecular origami in neuroscience: noncanonical DNA structures and functional nucleic acids in neural regulation and disease

**DOI:** 10.3389/fncir.2026.1893836

**Published:** 2026-10-26

**Authors:** Subhajit Roy, Arpit Kumar Pradhan, Ashwin Kumar Jainarayanan

**Affiliations:** 1 Center for Molecular Design and Biomimetics at the Biodesign Institute, Arizona State University, Tempe, AZ, United States; 2 Department of Physics, Arizona State University, Tempe, AZ, United States; 3 Department of Psychology, University of Michigan, Ann Arbor, MI, United States; 4 Department of Neurology and Neurological Sciences, Stanford Medicine, Stanford University, Stanford, CA, United States; 5 Kennedy Institute of Rheumatology, Nuffield Department of Orthopaedics, Rheumatology and Musculoskeletal Sciences (NDORMS), University of Oxford, Oxford, United Kingdom

**Keywords:** DNA aptamers, DNA origami, functional nucleic acids, glioma, G-quadruplexes, neuromodulation, neuro-oncology, noncanonical DNA structures

## Abstract

Gliomas are increasingly recognized as network diseases in which aberrant interactions between tumor cells, neurons, and immune populations drive hyperexcitability, seizures, cognitive dysfunction, and disease progression. Simultaneously, advances in structural biology and nucleic acid engineering have revealed DNA to be a dynamic molecular architecture capable of sensing, regulating, and modulating cellular states. In this review, we examine how noncanonical DNA structures and functional nucleic acids including Z-DNA, G-quadruplexes, cruciform structures, aptamers, and DNA origami systems, shape neural regulation and glioma associated circuit pathology. We discuss how activity dependent DNA conformational transitions influence transcriptional programs controlling neurotransmission, ion channel expression, synaptic signaling, metabolism, and inflammatory pathways in glioma cells and peritumoral neural networks. Z-DNA formation appears linked to rapid transcriptional activation and enhancer dynamics, whereas G-quadruplex rich regulatory regions modulate genes associated with excitatory signaling, cellular proliferation, and tumor progression. Cruciform DNA structures additionally interface with genome stability and DNA repair, creating mechanistic entry points for therapeutic intervention. Beyond endogenous DNA architectures, we highlight the emergence of aptamer based diagnostics and therapeutics capable of detecting neurotransmitters, cytokines, and metabolites associated with glioma induced network dysfunction. DNA origami platforms further enable programmable multivalent delivery systems for targeted transport of small molecules, oligonucleotides, CRISPR machinery, and ion-channel modulators across the neurovascular unit and tumor microenvironment. We also explore closed-loop neuromodulatory strategies in which electrophysiological signatures guide state dependent therapeutic actuation through nucleic acid based sensing and delivery systems. Finally, we discuss key translational considerations, including blood brain barrier delivery, nuclease stability, immunogenicity, biodistribution, manufacturing reproducibility, and integration with existing neuro-oncology and anti-seizure therapies. By bridging molecular origami, electrophysiology, and neurotechnology, this review proposes a framework for precision neuromedicine in glioma and broader neurological disease, where programmable nucleic acid architectures are coupled to the dynamic circuit states that drive pathology.

## Graphical abstract

**Figure:**
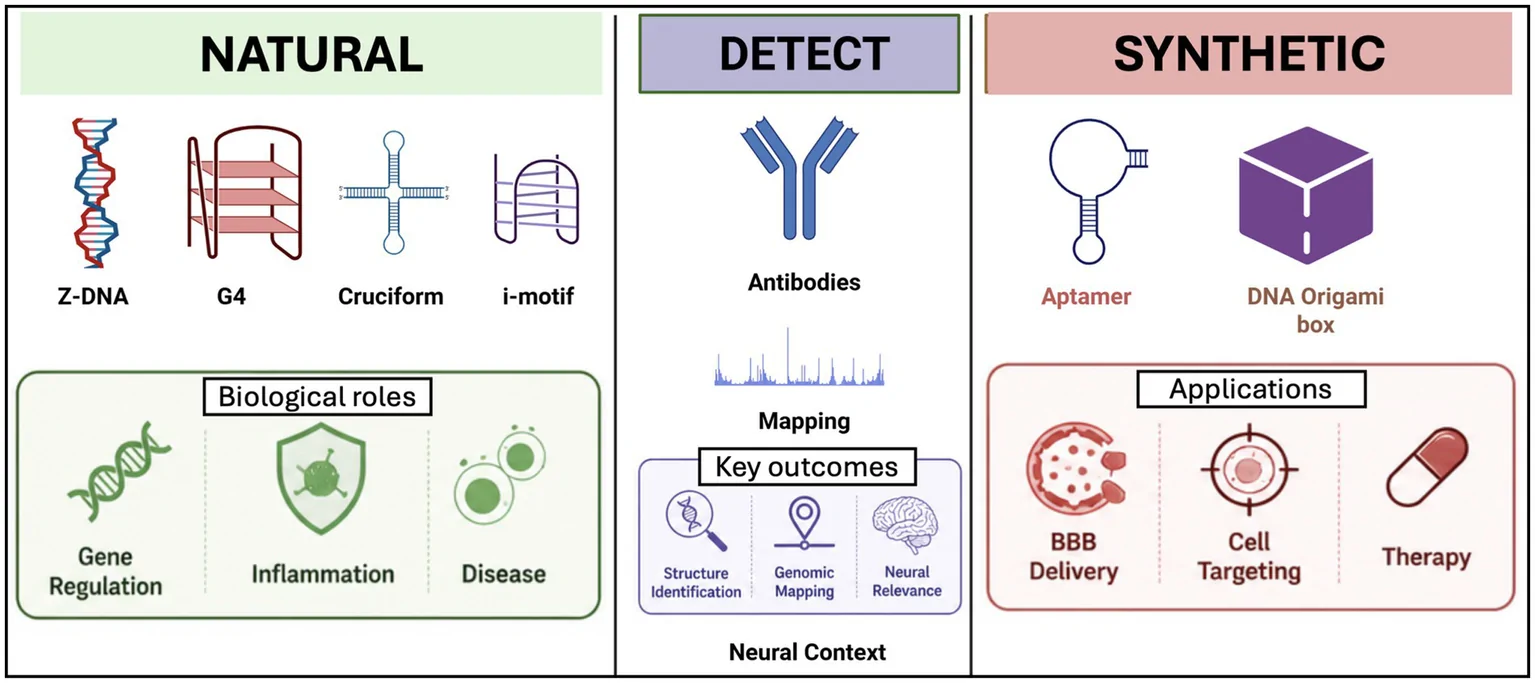

## Highlights

- Noncanonical DNA structures (Z-DNA, G-quadruplexes, cruciforms, and i-motifs) are functional, sequence-encoded regulatory elements, not curiosities, and constitute a fast, mechanical layer of gene regulation that complements the slower chemical marks of classical epigenetics.
- Their dysregulation is mechanistically linked to neurological disease, from repeat-expansion disorders (C9orf72, FMR1) and Alzheimer’s neuroinflammation to ATRX- and TERT-driven glioma, making them both biomarkers and therapeutic targets.
- i-Motifs add a pH-sensitive dimension that couples synaptic and tumor-microenvironment acidification to genome structure, though their physiological prevalence remains actively debated.
- Synthetic DNA aptamers (recognition) and DNA origami (architecture) enable programmable, reversible, cell-type-selective neuromodulation and delivery, but face nuclease stability, blood–brain-barrier, and manufacturing hurdles.
- The central unresolved challenge is selectivity and safety: structure-stabilizing ligands are promiscuous and double-edged, so locus-selective targeting and exhaustive, cell-type-resolved long-term safety profiling are prerequisites for clinical translation.

## 1 Introduction

The canonical Watson-Crick double helix has dominated our understanding of DNA structure since 1953, yet this elegant model represents only one of many conformations that DNA can adopt. Over the past four decades, structural biology has revealed that DNA is a remarkably polymorphic molecule capable of adopting alternative three-dimensional architectures in response to sequence composition, environmental conditions, and cellular mechanical forces. These noncanonical DNA structures including Z-DNA, G-quadruplexes, cruciforms, and other tertiary conformations are not merely biochemical curiosities but serve as functional regulatory elements that modulate gene expression, genome stability, and cellular responses to environmental stimuli.

The term “molecular origami” captures a profound analogy: just as the Japanese art of origami transforms a flat sheet of paper into complex three-dimensional forms through precise folding patterns, DNA sequences can fold into elaborate architectures that encode not only genetic information but also structural and regulatory functions. This concept extends from naturally occurring noncanonical structures to synthetically engineered DNA origami precisely designed nanostructures created by Paul Rothemund’s landmark 2006 scaffolded DNA origami method, building on Nadrian Seeman’s pioneering vision from 1980 that DNA could serve as a programmable building material for nanoscale construction.

In neuroscience, the concept of molecular origami takes on particular significance. The nervous system operates through exquisite spatiotemporal precision, where molecular events must be coordinated across scales ranging from individual synapses to whole-brain networks, and across timescales from milliseconds to decades. Neurons are post-mitotic cells that must maintain genomic integrity over the human lifespan while continuously adapting their transcriptional programs in response to experience. This creates unique vulnerabilities and requirements that make noncanonical DNA structures particularly relevant to neural function and dysfunction.

This review synthesizes emerging evidence that molecular origami both naturally occurring and synthetically designed plays crucial roles in neural biology. We examine how Z-DNA, G-quadruplexes, and cruciforms regulate neuronal gene expression and contribute to neurodegenerative pathology. We then explore how synthetic DNA aptamers and DNA nanotechnology are being harnessed to create next-generation diagnostics and therapeutics for neurological disorders. Throughout, we emphasize an integrated framework in which DNA tertiary structure serves as an additional layer of gene regulation, one that is particularly important in the unique cellular context of the nervous system.

## 2 Noncanonical DNA structures in neural biology

### 2.1 Z-DNA: a left-handed regulator of neuronal transcription

#### 2.1.1 Structural properties and formation mechanisms

Z-DNA represents a striking departure from the canonical B-form helix, adopting a left-handed configuration with a characteristic zigzag sugar-phosphate backbone that gives the structure its name. This alternative conformation forms most readily at alternating purine-pyrimidine sequences, particularly (CG)n and (CA/TG)n repeats, where the DNA undergoes a B-to-Z transition. Under relaxed physiological conditions, this transition is thermodynamically unfavorable. However, several factors can stabilize Z-DNA formation: negative supercoiling generated by transcriptional machinery, high salt concentrations, chemical modifications including cytosine methylation at CpG dinucleotides, and binding by specialized Z-DNA recognition proteins containing Zα domains (Pohl and Jovin, 1972; Run et al., 2026; Sahayasheela et al., 2025; Wang et al., 1979).

The molecular basis for Z-DNA’s unique structure lies in its alternating syn-anti glycosidic bond configuration, contrasting with B-DNA’s uniform anti conformation. This produces a deep, narrow minor groove and a convex major groove, creating a molecular surface topology fundamentally distinct from B-DNA. These structural differences enable specific protein recognition via Zα domains, found in proteins such as ADAR1 (adenosine deaminase acting on RNA 1) and ZBP1 (Z-DNA binding protein 1; Run et al., 2026; Sahayasheela et al., 2025; Wang et al., 1979).

#### 2.1.2 Z-DNA in transcriptional regulation and chromatin architecture

During active transcription, RNA polymerase II generates significant torsional stress as it unwinds the DNA helix ahead of the transcription bubble. This creates regions of negative supercoiling in the wake of the polymerase, providing the mechanical force that can drive B-to-Z transitions in susceptible sequences (Liu and Wang, 1987). Genome-wide mapping using Zα-based chromatin immunoprecipitation (ChIP-seq) strategies has revealed that Z-DNA-forming sites are enriched in promoter-proximal regions and correlate strongly with RNA polymerase II occupancy, high gene expression levels, and active chromatin marks, including H3K4me3 and H3K9ac (Shin et al., 2016). This distribution pattern suggests that Z-DNA formation is intimately linked to transcriptionally permissive chromatin states.

Z-DNA influences transcriptional regulation through multiple mechanisms. Because the incorporation of Z-DNA segments into nucleosomes is energetically unfavorable due to geometric incompatibilities, Z-DNA formation can drive nucleosome repositioning or exclusion from regulatory elements (Garner and Felsenfeld, 1987). This creates or reinforces open chromatin configurations that facilitate transcription factor access and polymerase engagement. Depending on genomic context and the complement of available regulatory proteins, Z-DNA has been associated with both transcriptional activation and repression, consistent with its role as a context-dependent structural switch rather than a simple activator or repressor (Sahayasheela et al., 2025; Shin et al., 2016).

A striking example of Z-DNA’s regulatory capacity comes from studies of thymic tolerance. Fang and colleagues demonstrated that AIRE (autoimmune regulator) target selection is influenced by promoter features associated with Z-DNA formation. Z-DNA-forming motifs were positively correlated with promoter double-strand break propensity and promoter poising a state characterized by accessible chromatin with pre-assembled transcriptional machinery ready for rapid activation. This work revealed how AIRE preferentially activates large sets of tissue-restricted antigens in medullary thymic epithelial cells, with Z-DNA serving as a cis-regulatory feature that flags AIRE-responsive loci for tolerance induction (Fang et al., 2024; Goldfarb and Abramson, 2024).

#### 2.1.3 Z-DNA dynamics in neural plasticity and memory

In the nervous system, Z-DNA functions as a dynamic, experience-regulated genomic feature rather than a static structural element. Marshall and colleagues provided compelling evidence for this concept through studies of fear conditioning and extinction in the mouse prefrontal cortex (Marshall et al., 2020). Following fear conditioning, Z-DNA levels increased at specific genomic loci. Remarkably, during fear extinction learning, these Z-DNA structures were actively resolved through recruitment of the RNA-editing enzyme ADAR1.

ADAR1 binding was associated with Z-DNA reduction at loci proximal to genes critical for synaptic function and neural circuit remodeling, including Nrxn3 (neurexin 3), Car5a (carbonic anhydrase 5a), Ints2 (integrator complex subunit 2), and Snap47 (synaptosome-associated protein 47; Marshall et al., 2020). Critically, blocking Z-DNA formation with doxorubicin or disrupting ADAR1 function impaired memory updating and behavioral flexibility without preventing initial fear acquisition or consolidation. This selective effect supports a model wherein Z-DNA formation serves as a reversible epigenetic mark that becomes functionally engaged during memory modification rather than during initial memory encoding (Marshall et al., 2020).

The proposed mechanism involves Z-DNA acting as a negative regulator of transcription at memory-relevant loci. When behavioral flexibility demands rapid transcriptional changes, ADAR1 resolves these Z-DNA structures, relieving transcriptional repression and enabling the coordinated gene expression programs necessary for memory updating. This represents a fundamentally dynamic use of DNA tertiary structure for neural information storage and retrieval, where the three-dimensional configuration of DNA itself encodes information about the behavioral state of the organism (Marshall et al., 2020).

#### 2.1.4 Z-DNA in neuroinflammation and neurodegeneration

Beyond transcriptional regulation, Z-DNA biology intersects with innate immunity through specialized Z-nucleic acid-sensing pathways. Z-DNA and the closely related Z-RNA are recognized by Zα-domain proteins, most notably ADAR1 (particularly the interferon-inducible p150 isoform) and ZBP1. Disrupted Zα-mediated recognition functions can trigger aberrant type I interferon responses, contributing to inflammatory pathologies including Aicardi-Goutières syndrome and bilateral striatal necrosis. In these conditions, ZBP1 can drive pathogenic interferon signaling when ADAR1 function is compromised, revealing a delicate balance in Z-nucleic acid homeostasis (Fang and Zhang, 2022).

Recent work has refined our understanding of Z-DNA’s role in Alzheimer’s disease, moving from simplistic causation to mechanistic insight. Song et al. demonstrated that in Alzheimer’s disease models, amyloid-*β*-associated oxidative stress promotes mitochondrial DNA fragmentation and induces a B-to-Z transition in released mtDNA fragments (Song et al., 2025). These Z-form mtDNA species are recognized by ZBP1, which recruits RIPK1 (receptor-interacting serine/threonine-protein kinase 1) to activate pro-inflammatory transcriptional mechanisms in microglia. Genetic deletion of Zbp1 or pharmacological inhibition of RIPK1 attenuated neuroinflammation and reduced pathological and behavioral deficits in AD mouse models, identifying a defined “Z-mtDNA-ZBP1-RIPK1” axis as a potential therapeutic target (Song et al., 2025). This work illustrates how these structures can serve as danger signals that couple cellular stress to inflammatory responses in the context of injury and neurodegenerative disorders (Sahayasheela et al., 2025; Song et al., 2025).

### 2.2 G-quadruplexes and cruciforms: dynamic tertiary structures in neurobiology

#### 2.2.1 Structural overview and formation principles

G-quadruplexes (G4s) and cruciform DNA represent two additional classes of noncanonical structures that play important roles in neural biology. G-quadruplexes form at guanine-rich sequences when a DNA or RNA strand folds such that four guanines hydrogen bond via Hoogsteen pairing (purine base rotates 180 degrees with reference to the helix axis) into planar G-tetrads, which stack to form stable four-stranded structures. These structures are stabilized by monovalent cations, particularly potassium, and can adopt multiple topologies (parallel, antiparallel, or hybrid) depending on strand orientation and loop configuration. This topological diversity makes G4s ideal as switchable structural microdomains that respond to changes in cellular ionic conditions and protein binding partners (Gellert et al., 1962; Sen and Gilbert, 1988; Williamson et al., 1989).

Cruciform DNA structures arise at inverted repeat or palindromic sequences in duplex DNA, particularly under conditions of negative supercoiling. Local strand separation enables each strand to fold back on itself to form intramolecular hairpins, producing a four-way junction reminiscent of a Holliday junction. This cruciform architecture creates a structural platform for protein recruitment and can serve as a substrate for structure-specific nucleases and resolvases (Lilley and Kemper, 1984; Mizuuchi et al., 1982; Panayotatos and Wells, 1981).

Both G4s and cruciforms are strongly influenced by DNA mechanics: torsional stress from transcription or replication, polymerase movement, local unwinding, and chromatin packaging all affect whether a sequence is permissive for structure formation, how long the structure persists, and which proteins can access it. This mechanical coupling means that these structures function as dynamic sensors of genomic activity rather than static architectural features (Brázda et al., 2011; Liu and Wang, 1987; Mizuuchi et al., 1982; Wang and Vasquez, 2023).

The structural and biophysical diversity of G-quadruplexes is considerable and directly governs their regulatory potential. A single guanine-rich sequence can fold into intramolecular or intermolecular species, and the relative orientation of the four strands defines the parallel, antiparallel, and hybrid (3 + 1) topologies, which differ in loop type (propeller, lateral, and diagonal) and in their syn/anti glycosidic torsion-angle distributions. These features determine groove widths and the accessibility of the terminal G-tetrad to proteins and small molecules. Thermodynamic stability scales with the number of stacked G-tetrads, is tuned by loop length and composition (shorter loops generally favoring parallel folds), and follows the cation hierarchy K^+^ > Na^+^ > Li^+^, reflecting the optimal fit of the dehydrated potassium ion within the central channel. In neurons, where intracellular K^+^ fluctuates sharply during action potentials, this cation dependence positions G4 folding equilibria as potential sensors of electrical activity. RNA G-quadruplexes, constrained by the ribose 2′-hydroxyl, almost invariably adopt parallel topologies and are typically more stable and less polymorphic than their DNA counterparts (Varshney et al., 2020).

#### 2.2.2 Physiological functions in transcription and chromatin organization

G-quadruplexes are increasingly recognized not merely as transcriptional roadblocks but as sophisticated regulatory elements that modulate gene expression through multiple mechanisms. G4 formation can alter transcription factor occupancy by competing for DNA binding sites or by creating structural barriers. They influence polymerase kinetics, with some polymerases pausing or stalling at these structures while specialized helicases resolve them to allow progression. G4s also affect nucleosome positioning and local epigenetic environments, functioning as shape-encoded regulatory elements that produce context-specific outcomes depending on when they form and which binding partners are available (Besnard et al., 2012; Biffi et al., 2013; Chambers et al., 2015; Chen et al., 2018; Hänsel-Hertsch et al., 2016; Robinson et al., 2021).

Cruciform structures similarly act as regulatory DNA shapes. Because a cruciform is a four-way junction embedded in duplex DNA, it can behave as a structural landmark for architectural proteins, topoisomerases, and chromatin regulators, supporting roles in gene expression control and local chromatin organization when properly regulated. The functional significance of both structure types extends to DNA replication, where G4 motifs are enriched near many metazoan replication origins and contribute to origin firing efficiency (Besnard et al., 2012; Brázda et al., 2011; Mizuuchi et al., 1982; Prorok et al., 2019).

A particularly instructive example of productive G4 function comes from adaptive immunity. During immunoglobulin heavy-chain (IgH) class switch recombination in B cells, switch region sequences produce G-rich transcripts that fold into RNA G-quadruplexes. The RNA helicase DDX1 binds these G4 RNAs and converts them into R-loops (RNA–DNA hybrids) over switch regions, helping to target activation-induced cytidine deaminase (AID) and organize the CSR machinery (de Almeida et al., 2018). Disrupting DDX1 function or chemically stabilizing G4 RNA perturbs R-loop formation and reduces CSR efficiency. Although this occurs in B cells rather than neurons, it demonstrates a general principle relevant throughout the nervous system: cells can deliberately interconvert structural states (G4 ↔ R-loop) to direct enzymatic outcomes, a logic that appears in neuronal gene regulation and disease.

#### 2.2.3 G-quadruplexes as activity-dependent regulatory switches in neurons

The most direct evidence linking G4 structures to neurophysiology comes from studies showing that G4-DNA accumulates in neurons in an experience-dependent manner and is required for proper temporal regulation of learning and memory. In mice, G4-DNA dynamics were connected to regulated silencing and activation of memory-relevant genes. Targeted manipulation of G4 resolution achieved by recruiting the G4 helicase DHX36 to specific loci was sufficient to perturb fear extinction memory, supporting the idea that G4 formation and resolution operate as a molecular timing device for neuronal gene expression programs (Chen et al., 2018; Marshall et al., 2024).

This work positions DNA tertiary structure within mainstream neuroepigenetics, alongside histone modifications and transcription factor networks. Activity-dependent transcription is not solely about chromatin marks and transcription factors but also involves regulated DNA folding states that shape transcriptional trajectories. Furthermore, G4 structures in RNA play crucial roles in neuronal biology. The Fragile X Messenger Ribonucleoprotein protein (FMRP) promotes RNA localization to neuronal projections via interactions between its RGG domain and RNA G-quadruplex sequences enriched in 3′ untranslated regions of localized transcripts. This links G4 structure to subcellular RNA targeting and thereby to synaptic function and plasticity, illustrating how the same structural motif can regulate both transcription (via DNA G4s) and translation/localization (via RNA G4s).

#### 2.2.4 Pathological roles in neurodegeneration

When G4s and cruciforms persist inappropriately or accumulate at vulnerable loci, they can drive transcriptional dysregulation and genome instability (Brázda et al., 2011; Wang and Vasquez, 2023). In Alzheimer’s disease (AD) neurons, excessive G4 structures arising from specific genomic contexts have been associated with altered neuronal gene expression and splicing patterns (Hanna et al., 2021). A key insight is that chromatin repression mechanisms normally limit excessive G4 formation, implying that epigenetic failure can indirectly produce a structural DNA failure (Hanna et al., 2021). This supports a disease model wherein G4 formation is not merely a downstream marker but actively participates in transcriptional disruption when the chromatin-structure equilibrium is perturbed (Hanna et al., 2021).

Repeat expansion disorders provide particularly clear examples of G4-mediated pathology. The C9ORF72 GGGGCC (G4C2) hexanucleotide repeat expansion the most common known genetic cause of amyotrophic lateral sclerosis (ALS) and frontotemporal dementia (FTD) involves expanded repeat RNAs that adopt G-quadruplex conformations (Conlon et al., 2016). In patient brain tissue, repeat RNA forms G-quadruplex inclusions that sequester RNA-binding proteins, notably heterogeneous nuclear ribonucleoprotein H (hnRNP H), disrupting splicing regulation. The repeat expansion also drives other toxic pathways including repeat-associated non-ATG translation (Mori et al., 2013; Zu et al., 2011), but the G4 scaffold provides a concrete molecular basis for selective RNA-binding protein sequestration and nuclear inclusion formation (Conlon et al., 2016).

Similar mechanisms operate in fragile X-associated tremor/ataxia syndrome (FXTAS), caused by CGG repeat expansions in the FMR1 premutation range (Asamitsu et al., 2021). CGG repeat RNA can form G-quadruplex structures that interact with FMRpolyG (a toxic protein produced via repeat-associated non-ATG translation), promoting aggregation and neuronal dysfunction with evidence for propagation of pathology across neural networks (Asamitsu et al., 2021; Zu et al., 2011). In CANVAS (cerebellar ataxia, neuropathy, vestibular areflexia syndrome), expansion of AAGGG pentanucleotide repeats in RFC1 produces DNA and RNA parallel G-quadruplexes (Wang et al., 2024). Structural characterization reveals that these G4 structures cause replication stalling and reduced gene expression in a repeat-length-dependent manner, providing a mechanistic link between structure formation and neurodegenerative phenotype (Wang et al., 2024; Figure 1).

**Figure 1:**
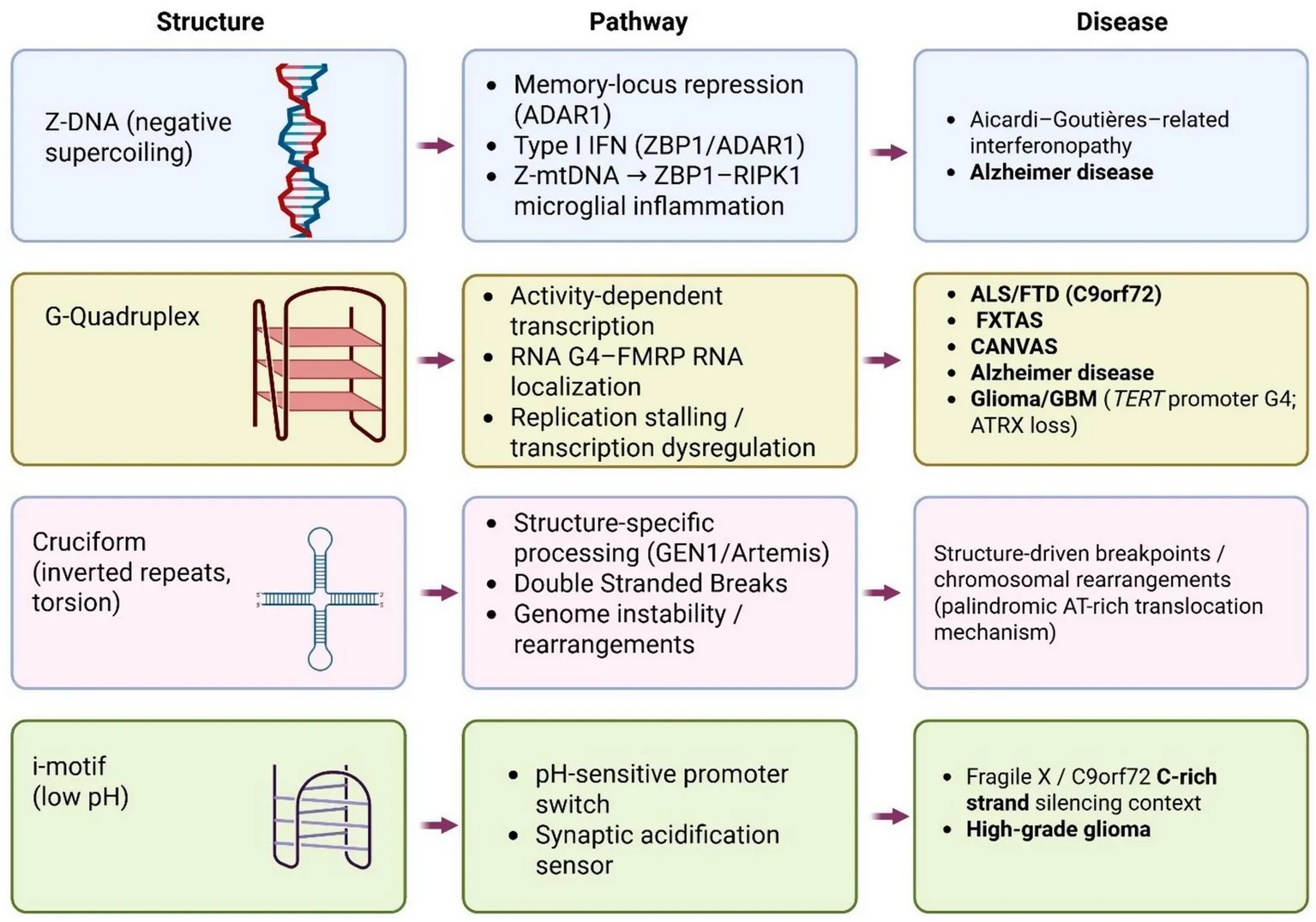
Noncanonical DNA structures as dynamic regulatory elements in neural biology and disease. Canonical B-DNA can adopt alternative conformations—Z-DNA, G-quadruplexes (G4s), cruciforms, and i-motifs—under distinct sequence contexts and biophysical cues. Z-DNA, favored by negative supercoiling, contributes to memory-locus transcriptional repression (resolved by ADAR1) and innate immune signaling via ZBP1/ADAR1 Type I interferon pathways, including Z-form mtDNA–driven ZBP1–RIPK1 microglial inflammation linked to Alzheimer disease and Aicardi–Goutières–related interferonopathy. G4s form at guanine-rich tracts and are stabilized by monovalent cations (notably K^+^); they support activity-dependent transcription and RNA G4–FMRP–mediated RNA localization, but can also cause replication stalling and transcriptional dysregulation, with associations to ALS/FTD (C9orf72), FXTAS, CANVAS, Alzheimer disease, and glioma/GBM (including *TERT* promoter G4s and ATRX loss). Cruciforms extrude from inverted repeats under torsional stress and are processed by structure-specific factors (e.g., GEN1/Artemis), promoting double-strand breaks, genome instability, and rearrangements. i-motifs fold in cytosine-rich sequences at low pH and act as pH-sensitive promoter switches and synaptic acidification sensors, with links to Fragile X/C9orf72 C-rich strand silencing and high-grade glioma. Dysregulation or persistence of these structures can converge on neuronal dysfunction, neurodegeneration, and malignancy.

Cruciform DNA contributes to neurological disease primarily through genome instability mechanisms (Brázda et al., 2011). While less often explicitly named in neurodegeneration literature compared to G4s, cruciforms are deeply relevant through their role as fragile, structure-driven breakpoints (Brázda et al., 2011; Kurahashi and Emanuel, 2001). A canonical example is the recurrent constitutional t(11;22) translocation, where breakpoints map to palindromic AT-rich repeats consistent with hairpin/cruciform extrusion and double-strand break formation at the palindrome center (Kurahashi and Emanuel, 2001). Mechanistically, cruciform DNA can be processed by enzymes normally acting in other pathways: GEN1-mediated resolution at the four-way junction followed by Artemis opening of hairpin ends produces palindrome-mediated chromosomal translocations (Inagaki et al., 2013). For neuroscience, the key conceptual link is that cruciform-prone palindromes convert torsional stress into defined DNA breakage routes, with germline rearrangements and somatic genome instability having profound downstream neurological consequences (Brázda et al., 2011; Inagaki et al., 2013; Kurahashi and Emanuel, 2001).

G-quadruplexes are also strongly implicated in brain tumors, where they contribute to malignancy through several converging mechanisms. In glioblastoma, recurrent mutations in the promoter of telomerase reverse transcriptase (TERT; e.g., C228T and C250T) generate highly stable G-quadruplex topologies that alter transcription-factor binding and drive aberrant TERT reactivation and cellular immortalization (Wang and Vasquez, 2023). A second axis links G4 biology to genome instability: inactivating mutations in the chromatin remodeler ATRX, which normally helps resolve G4 structures, lead to reversible G4 accumulation at GC-rich loci, replication-fork stalling, and DNA damage that drives the copy-number alterations characteristic of ATRX-deficient malignant glioma (Wang et al., 2019). Because such tumors depend on G4 resolution and recombination-based repair, they are selectively vulnerable to pharmacological G4 stabilization. G-quadruplexes also act at the RNA level: in glioblastoma, the RNA-binding proteins hnRNP H and hnRNP F, together with the helicase DHX36, remodel RNA G-quadruplexes in the untranslated regions of DNA damage response transcripts to keep them translationally permissive, thereby elevating DNA-repair capacity and contributing directly to chemo- and radio-resistance; small-molecule rG4 stabilization phenocopies hnRNP H/F depletion and re-sensitizes tumor cells to genotoxic therapy (Herviou et al., 2020). The first-line alkylating agent temozolomide compounds this dependency, as its preferential mutagenesis of guanine residues remodels G4 motifs and G-rich splice sites in drug-resistant gliomas.

### 2.3 I-motifs: pH-sensitive regulators of the neural genome

#### 2.3.1 Structural configuration and stabilization

The intercalated motif (i-motif) is a four-stranded structure that folds within cytosine-rich sequences, often complementary to G-quadruplex-forming strands. It is built from hemi-protonated cytosine-cytosine (C: C^+^) base pairs, which intercalate in an antiparallel orientation and require protonation of cytosine N3, making i-motif formation acutely pH-sensitive. Once dismissed as an *in vitro* curiosity forming only under acidic conditions, i-motifs are now known to form at physiological pH under negative supercoiling, molecular crowding, and protein binding. Development of the i-motif-specific antibody iMab enabled their visualization in the nuclei of living human cells, revealing dynamic, cell-cycle-dependent folding that peaks at the G1/S transition (Zeraati et al., 2018).

#### 2.3.2 Regulatory roles, chaperones, and repeat-expansion disorders

Like G-quadruplexes, i-motifs are enriched at promoters and transcription start sites, where they act as structural switches: i-motif formation in the promoter of an oncogene such as MYC or a growth factor such as PDGFRB can repress or activate transcription depending on the recruited proteins (Kang et al., 2014; Kendrick et al., 2014). Because these structures are transient and pH-sensitive, their formation and resolution *in vivo* are actively managed by protein chaperones. In addition to stabilizing factors such as hnRNP LL, hnRNP K, and ATRX, poly(rC)-binding protein 1 (PCBP1) selectively binds and unfolds i-motifs to prime these loci for replication and transcription; its occupancy peaks at G1/S, and its loss leads to i-motif accumulation, cell-cycle arrest, and genomic instability. This regulated equilibrium is directly relevant to neurological repeat-expansion disorders: the cytosine-rich strands complementary to G4-forming expansions, such as the CCG strand of the FMR1 CGG repeat in fragile X syndrome and the C-rich strand of the C9orf72 expansion in ALS/FTD, can fold into stable i-motifs that nucleate heterochromatin and contribute to allele-specific silencing.

### 2.4 pH sensitivity, synaptic acidification, and glioma

The pH sensitivity of the i-motif provides a distinctive bridge between neuronal activity and genomic structural dynamics. Synaptic transmission is accompanied by localized acidification, and such microenvironmental pH shifts are sufficient to promote i-motif folding in C-rich promoters of activity-dependent genes, providing a structural sensor of neural activity. Pathological, sustained acidosis, as occurs in ischemia, epilepsy, or neuroinflammation, can lock these structures in place and disrupt transcription. This principle is amplified in high-grade gliomas, where the Warburg effect produces a chronically acidic extracellular microenvironment that can stabilize i-motifs in the promoters of oncogenes and angiogenic regulators such as VEGFA, BCL2, and TERT, making i-motif-directed small molecules an attractive, microenvironment-selective therapeutic strategy for brain tumors (Table 1).

**Table 1:** Comparison of the major noncanonical DNA structures relevant to the nervous system.

Structure	Sequence motif	Key stabilizing factors	Representative neurological association
Z-DNA	Alternating purine–pyrimidine repeats, esp. (CG)n and (CA/TG)n	Negative supercoiling; 5-methylcytosine; Zα-domain proteins (ADAR1, ZBP1)	Aicardi–Goutières syndrome; Alzheimer disease (Z-mtDNA → ZBP1–RIPK1)
G-quadruplex	G-rich tracts, typically G ≥ 3 N1–7 (DNA or RNA)	K^+^ (and other monovalent cations); molecular crowding; G4 ligands	ALS/FTD (C9orf72); FXTAS; CANVAS; Alzheimer disease; glioma/GBM (TERT, ATRX)
Cruciform	Inverted repeats/palindromes	Negative supercoiling; junction-binding/structure-specific proteins (e.g., GEN1, Artemis)	Genome instability; t(11;22) and related palindromic breakpoints
i-Motif	C-rich tracts complementary to G4 strands (C:C^+^ pairs)	Acidic pH; negative supercoiling; molecular crowding; stabilizers (hnRNP LL/K, ATRX)*	High-grade glioma (tumor acidosis); Fragile X / C9orf72 C-rich silencing; synaptic pH-linked regulation

^*^PCBP1 binds and unfolds i-motifs (resolution chaperone) and is therefore not listed as a stabilizing factor.

### 2.5 Comparison with classical epigenetic mechanisms

Noncanonical DNA structures are best understood alongside the classical epigenetic machinery of DNA methylation, histone modifications, and ATP-dependent chromatin remodeling. Classical marks are enzymatic and comparatively slow, deposited by writers (e.g., DNMTs, HATs) and interpreted by readers (e.g., MeCP2) over minutes to days to encode stable cellular memory. In contrast, Z-DNA, G-quadruplexes, and i-motifs are mechanical and thermodynamic switches driven largely by the negative superhelical tension generated behind moving RNA polymerases; they form and relax on sub-second timescales, providing a real-time, transient regulatory layer. The two systems also intersect physically: the rigid geometries of these structures are incompatible with nucleosome wrapping, so their formation excludes nucleosomes and creates open, accessible chromatin upstream of remodeling complexes. Finally, whereas chemical marks can be placed genome-wide depending on enzyme recruitment, structural folding is strictly sequence-encoded at defined hotspots (CG-repeats, G-runs, C-runs, inverted repeats).

Rather than a parallel system, the two layers regulate one another bidirectionally. 5-methylcytosine alters the stability of both Z-DNA and i-motifs, while structure formation can in turn shield a locus from, or expose it to, the methylation machinery. R-loops, three-stranded DNA/RNA hybrids whose displaced strand frequently folds into a G4 or i-motif, illustrate this integration in the developing brain: they are abundant at unmethylated CpG-island promoters of genes destined for later upregulation, where they poise transcription through Polycomb and histone-deacetylase complexes, and their experimental removal triggers premature neuronal gene expression and disrupts differentiation. R-loops also shape the methylome by obstructing maintenance methylation and recruiting TET enzymes, coupling a transient structural mark to the stable chemical marks that define neural identity.

### 2.6 Methodologies for detecting noncanonical DNA structures in neuronal systems

Establishing the functional relevance of noncanonical DNA in the brain has depended on tools to detect, map, and characterize these structures. Structure-specific antibodies have been pivotal: BG4 visualizes G-quadruplexes in human and rodent brain slices, iMab reports i-motif formation in living nuclei, and the Zα domain of ADAR1/ZBP1 or the Z22 antibody detects Z-DNA. Coupled to structure-specific affinity reagents, chromatin immunoprecipitation sequencing (ChIP-seq) and Cleavage Under Targets and Tagmentation (CUT&Tag) map these structures genome-wide; BG4 CUT&Tag in particular offers higher resolution and far lower input than ChIP-seq (as few as 10^5^–10^6^ cells), bringing rare neuronal populations, organoids, and post-mortem tissue within reach. Cell-type-specific nuclei sorting and emerging single-cell G4 CUT&Tag now resolve structural landscapes across the heterogeneous cell types of the brain, which is essential for interpreting repeat-expansion loci such as the C9orf72 G4-forming repeat in motor neurons.

Complementary biophysical methods provide structural and dynamic detail: circular dichroism and NMR spectroscopy resolve topology and stability, while single-molecule FRET tracks folding-unfolding kinetics in real time. Small-molecule fluorescent probes (e.g., pyridostatin and Thioflavin-T derivatives for G4s, BMVC-type compounds for i-motifs) enable live-cell monitoring. Finally, computational tools scan neural genomes for structural propensity, ranging from motif scanners (QGRS Mapper, Quadparser) and the score-based G4Hunter to machine-learning predictors of cell-type-specific activity (DeepG4), stability (Quadron), and i-motif formation (iM-Seeker); a reagent-free alternative infers structure genome-wide from the sequencing-error signatures that noncanonical folds impose on DNA polymerases, and can be applied retrospectively to existing datasets from Alzheimer’s, Parkinson’s, and ALS brains.

## 3 Synthetic DNA aptamers and functional nucleic acids for neuromodulation

### 3.1 DNA aptamers as programmable molecular recognition elements

While the previous sections examined naturally occurring DNA tertiary structures, synthetic biology enables the deliberate design of nucleic acid sequences that fold into precise three-dimensional shapes with programmable functions. DNA aptamers represent a particularly powerful class of such molecules: short single-stranded DNA oligonucleotides (typically 20–100 nucleotides) that fold into defined tertiary structures capable of binding specific molecular targets with high affinity and specificity. Unlike antibodies, which are produced biologically and require animal immunization or phage display, DNA aptamers are selected *in vitro* through an iterative process called SELEX (Systematic Evolution of Ligands by EXponential enrichment).

The SELEX process begins with a randomized library of DNA sequences (typically 10^14^–10^15^ unique sequences) and enriches those that bind to a target through iterative rounds of binding, separation, and amplification. For neurological applications, three SELEX variants are particularly relevant: (1) protein-target SELEX, where aptamers are selected against purified proteins relevant to neurodegeneration; (2) cell-SELEX, where selection occurs against intact cells, capturing native membrane contexts including glycosylation patterns, receptor clustering, and microenvironmental effects; and (3) tissue or *in vivo* SELEX, enabling specificity even when the molecular target is unknown.

DNA aptamers offer several advantages for neurological applications compared to protein-based therapeutics. They are chemically synthesized, enabling precise quality control and chemical modifications to enhance stability. They can be designed to be reversible through addition of complementary “antidote” strands that disrupt the active fold—a safety feature particularly attractive when modulating excitable neural tissue. They have minimal immunogenicity compared to antibodies. They can penetrate tissue more effectively due to their small size. And perhaps most importantly, they follow a “sequence-to-function” design logic where the same chemical alphabet that encodes genetic information also encodes structure, multivalency, and reversible on/off behavior (Figure 2).

**Figure 2:**
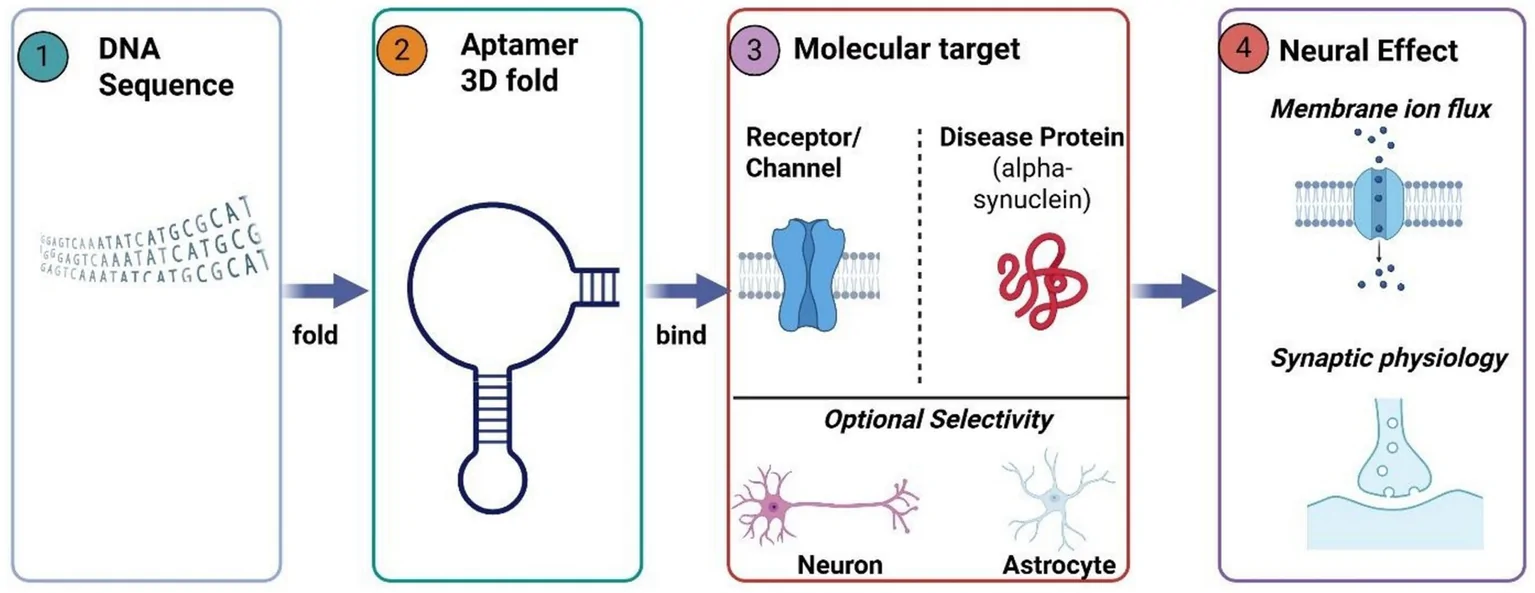
Sequence-to-function logic for aptamer neuromodulation. DNA sequence encodes folding into a defined three-dimensional aptamer structure that enables selective binding to molecular targets, including membrane receptors/channels or disease-associated proteins (e.g., α-synuclein). Optional cell-type selectivity (e.g., neurons versus astrocytes) further refines target engagement. Bound aptamers can alter membrane ion flux and signaling, ultimately modulating synaptic physiology. The schematic highlights the programmable “sequence → fold → bind → function” paradigm underlying aptamer-based neuromodulation.

It is worth distinguishing the two DNA-based platforms invoked throughout this review, as they operate at different scales. A DNA aptamer is a single short oligonucleotide (tens of nucleotides) whose sequence folds into one binding-competent shape; it functions as a molecular-recognition element, the nucleic-acid analogue of an antibody paratope. DNA origami, by contrast, uses a long scaffold strand folded by hundreds of short “staple” strands into a defined, addressable nanostructure (tens of nanometers), functioning less as a single binder than as a programmable chassis onto which aptamers, drugs, and stimuli-responsive gates can be positioned with nanometer precision. Aptamers thus supply recognition, whereas origami supplies architecture, and the two are frequently combined.

### 3.2 Aptamers targeting neurodegenerative disease proteins

#### 3.2.1 α-synuclein aptamers for Parkinson’s disease

A major DNA-forward strategy for neuromodulation uses aptamers as synthetic ligands targeting pathological proteins. In Parkinson’s disease, DNA aptamers selected against α-synuclein have been reported to bind in the nanomolar range, reduce aggregation *in vitro* and in cellular models, and promote intracellular clearance pathways while rescuing cellular dysfunction phenotypes linked to α-synuclein overexpression (McConnell et al., 2024; Zheng et al., 2018). Conceptually, this represents neuromodulation at the molecular source: rather than adjusting downstream neuronal firing pharmacologically, the DNA ligand directly perturbs a disease-driving protein state (misfolding and aggregation) that ultimately degrades synaptic function, mitochondrial health, and neuronal viability.

#### 3.2.2 Amyloid-*β* and tau aptamers for Alzheimer’s disease

Similarly, DNA aptamers designed against BACE1 (β-site amyloid precursor protein cleaving enzyme 1) have been reported to inhibit BACE1 activity, reduce amyloid-β pathogenesis, and rescue neuronal deficiencies in cultured systems (Xiang et al., 2019), suggesting a route to reshape amyloidogenic pressure using nucleic acid ligands rather than traditional small-molecule enzyme inhibitors. More broadly, aptamers targeting amyloidogenic proteins are being explored not only as simple binders but as aggregation-state-selective tools (Murakami et al., 2022)—an important distinction in neurodegeneration where toxic oligomeric intermediates can matter more than total protein abundance.

A particularly compelling direction couples aptamer recognition to cellular effector functions, converting a binder into an active clearance mechanism. Researchers have constructed aptamer-armed monocytes designed to recognize extracellular tau and leverage innate phagocytic capacity, reporting reduced tau burden and reduced neuroinflammation in mouse models (Zhuo et al., 2025). This illustrates how DNA recognition elements can redirect immune cell behavior toward neurotherapeutic goals. In parallel, functional selection approaches enrich DNA aptamers not merely for binding tau but for binding modes that interfere with pathological oligomerization pathways, moving closer to the therapeutic objective than affinity alone.

#### 3.2.3 Aptamers for ionotropic receptor modulation

Beyond targeting pathological proteins, DNA and RNA aptamers have been developed against key neuronal receptors. RNA aptamers that antagonize NMDA receptors have been characterized, demonstrating that nucleic acid ligands can modulate excitatory neurotransmission (Lee et al., 2014). Similarly, long aptamers blocking AMPA receptors and short aptamers blocking both AMPA and kainate receptors have been identified and characterized (Jaremko et al., 2017). Preclinical testing of AMPA receptor RNA aptamers in an ALS mouse model has evaluated therapeutic efficacy and safety, illustrating the potential for aptamer-based modulation of synaptic transmission in neurodegenerative contexts where excitotoxicity contributes to pathology.

### 3.3 Blood–brain barrier penetration and brain-targeted delivery

A fundamental challenge for any CNS therapeutic is crossing the blood–brain barrier (BBB), a specialized endothelial barrier that restricts the passage of most molecules into the brain parenchyma. DNA aptamer strategies increasingly focus on BBB addressing as a prerequisite for neurological applications. One approach couples DNA recognition to nanomaterial pharmacokinetics: transferrin receptor-targeting DNA aptamers improve BBB penetration of protein spherical nucleic acids (dense nucleic acid shells presented on protein cores; Kusmierz et al., 2022), demonstrating how aptamers can serve as receptor-mediated entry keys while the larger architecture governs circulation and stability.

A complementary strategy uses endogenous biological carriers. ATP-responsive DNA aptamer sensors packaged into brain cell-derived exosomes achieve BBB passage and enable *in vivo* brain readouts, including differential ATP signals across brain regions in Alzheimer’s disease mouse models (Banik et al., 2024). This demonstrates how DNA devices can be paired with biological vectors to create tissue-selective sensing and, in principle, closed-loop modulation. Once DNA constructs reach the brain, the bottleneck becomes which cell populations internalize them and whether they escape degradative trafficking.

The dominant non-invasive route exploited by these platforms is receptor-mediated transcytosis (RMT), in which a ligand engages a receptor on the luminal endothelial membrane, triggering endocytosis, transcellular vesicular transport, and abluminal release. The transferrin receptor (TfR) and low-density lipoprotein receptor-related protein 1 (LRP1) are the most exploited RMT targets, and SELEX-derived DNA aptamers provide programmable, high-affinity keys to these pathways. Conjugation strategies can be strikingly effective: a TfR-targeting aptamer tethered to a lipid moiety has been reported to deliver DNA oligonucleotides to the mouse brain with brain-to-serum ratios as high as 6.5, while transferrin aptamer-functionalized protein spherical nucleic acids markedly increase brain accumulation. Aptamer engineering, such as truncation to remove nonfunctional domains, further tunes binding kinetics and endosomal release to avoid entrapment within the endothelium.

Beyond the choice of ligand, the physical form of the DNA construct shapes brain access. Systematic screening of wireframe DNA origami geometries indicates that compact, cube-like nanostructures show favorable brain distribution, an effect linked to the serum protein corona that mediates endothelial uptake, and intact three-dimensional frameworks outperform single strands in transcytosis. Pharmacokinetic optimization through PEGylation, cholesterol conjugation, and 2′-sugar modifications extends circulation and nuclease resistance, widening the window for receptor engagement. Where receptor-mediated routes are insufficient, adjuncts can be layered on: microbubble-assisted focused ultrasound transiently and focally opens tight junctions to admit carriers at defined sites such as tumor margins or amyloid-rich regions; intranasal administration bypasses the vasculature via olfactory and trigeminal pathways; and cell-mediated “Trojan horse” transport hijacks the tropism of monocytes, macrophages, or neural stem cells for diseased brain tissue. Together these move the field from single-route targeting toward integrated, stimulus-aware delivery designs.

Recent designs integrate multiple specificity layers. Transferrin receptor aptamer-programmed DNA nanoflowers (assembled via rolling-circle amplification) enable BBB penetration followed by intracellular release behavior supporting neuroinflammatory intervention, including delivery of antisense oligonucleotide strategies targeting NLRP3 signaling (Kong et al., 2025). The design logic demonstrates tissue-specific modulation through layered targeting: vascular targeting (BBB) → brain enrichment → cell-level processing (triggered disassembly/escape) → pathway-level silencing, rather than relying on a single targeting step.

### 3.4 Cell-type specificity and tissue-selective modulation

Precision neuromodulation requires not only crossing the BBB but also engaging specific cell types. A central challenge is that the same receptor family, signaling pathway, or inflammatory cascade can have different consequences depending on whether it acts in neurons, astrocytes, or microglia, or in one brain region versus another. DNA aptamers are increasingly used as programmable specificity layers that recognize defined cell-surface signatures, bias uptake into particular cell types, or restrict payload activation until tissue-appropriate molecular triggers are encountered.

While protein-target SELEX yields binders against purified targets, cell-SELEX and tissue/*in vivo* selection enrich DNA aptamers directly against intact cells or complex tissues, capturing native membrane contexts and enabling specificity even when the molecular target is initially unknown. DNA aptamers selected for affinity to neuroblastoma cell models and screened for biological activity demonstrate binding across differentiated cells and human stem cell-derived neuronal lineages. This pipeline treats DNA not only as a targeting ligand but as a platform for discovering bioactive extracellular binders that can influence neurite-related or stress-response phenotypes in a cell-type-dependent way.

Cell-type selectivity becomes tangible when aptamers discriminate between major CNS cell classes. Anti-astrocyte DNA aptamers identified by cell-SELEX have been engineered into trivalent Y-shaped constructs to increase avidity, enabling rapid astrocyte-specific endocytosis and clearer discrimination between astrocytes and neurons (Oh et al., 2025). The trivalent architecture converts modest monovalent binding into robust, selective uptake a general design rule for tissue specificity applicable when target epitopes are moderately abundant on one cell type but sparse on another. This is directly relevant for glia-focused therapies where limiting off-target neuronal uptake is critical for tuning astrocyte inflammatory or metabolic states.

### 3.5 DNA nanotechnology for direct neural modulation

DNA nanotechnology extends the concept of molecular origami from recognition elements to functional devices that directly influence ion transport and membrane potential. A striking example is the development of reversible, photothermal-gated DNA nanochannels integrated with Janus nanoparticles. Near-infrared light triggers conformational changes that gate ion transport, rapidly modulating neuronal excitability (Li et al., 2026). Proof-of-concept demonstrations include restoration of pain sensation in NaV1.7-knockout mice upon brief laser irradiation, positioning DNA nanochannels as a non-genetic neuromodulation modality with external spatiotemporal control.

This approach sits within growing work on DNA nanopores and synthetic DNA ion channels, where DNA origami and related designs provide precise control over pore geometry, functionalization, and stimulus responsiveness. Key engineering challenges include structural fluctuations and leakage that must be solved for robust physiological use. Even when DNA does not act as a channel itself, it can modulate signaling by controlling nanoscale receptor organization—a level of regulation central to synaptic biology. DNA origami scaffolds can set ligand valency and spacing with nanometer precision, tuning receptor clustering and downstream signaling efficiency (Chen et al., 2025; Zhang et al., 2017). This approach is increasingly viewed as a general platform for interrogating how spatial organization encodes function, with direct translational relevance: synaptic strength and plasticity depend on receptor nanodomains, co-receptor geometry, and local cooperative interactions that DNA scaffolds can probe and potentially steer.

DNA also contributes to neuromodulation through sensing-enabled therapy. DNA aptamers serving as molecular recognition elements for neurotransmitters or neuromodulators can be coupled to electrochemical readouts, creating dopamine-detection aptasensors as proof-of-concept systems. Such sensors are prerequisites for closed-loop neuromodulation systems that measure a molecular state (e.g., neurotransmitter level) and adjust an intervention in real time. While many sensors remain in their early stages, they represent strategic capabilities for future autonomous therapeutic systems.

## 4 Challenges, integration with emerging technologies, and future prospects

### 4.1 Stability, delivery, and immunogenicity

Despite tremendous promise, translating DNA-based therapeutics and diagnostics into clinical reality faces several fundamental challenges. Nuclease degradation remains a primary concern, as unmodified DNA aptamers are rapidly degraded by serum nucleases. Chemical modifications including 2’-O-methyl ribose substitutions, locked nucleic acids (LNAs), phosphorothioate linkages, and 3′-end capping can substantially improve stability. However, modifications must be balanced against potential impacts on binding affinity, specificity, and folding kinetics.

Delivery to the CNS presents multiple barriers beyond the BBB. Achieving cell-type-specific uptake requires not only surface targeting but also appropriate endosomal escape mechanisms to prevent lysosomal degradation. For DNA nanostructures like origami, maintaining structural integrity in biological fluids while achieving sufficient circulation time and avoiding rapid renal clearance requires careful engineering of size, shape, and surface chemistry. The field is moving from single-mechanism targeting toward integrated designs that combine vascular addressing, cellular uptake signals, and intracellular trafficking cues in stoichiometrically controlled combinations.

Immunogenicity is context-dependent. While DNA aptamers generally show lower immunogenicity than protein biologics, certain DNA sequences, particularly those containing unmethylated CpG motifs can activate toll-like receptor 9 (TLR9) signaling and trigger innate immune responses. This can be advantageous for vaccine adjuvants but problematic for chronic neurological applications. Sequence optimization to avoid immunostimulatory motifs, along with chemical modifications that reduce TLR9 activation, are active areas of development. The challenge is particularly acute for long-term neurological applications where repeated dosing may be required.

Beyond stability and immunogenicity, the safety of targeting noncanonical DNA structures deserves explicit attention, because the very ligands used to stabilize them are structurally promiscuous. Most G4 ligands are large planar aromatics that stack on the conserved terminal G-tetrad, a binding mode largely indifferent to genomic context; a single compound can therefore stabilize G4s at oncogene promoters, telomeres, replication origins, and ribosomal DNA simultaneously, and can cross-react with RNA G-quadruplexes and R-loops. These off-target activities bear directly on whether such approaches perturb normal neuronal function and genomic stability. In cells with intact repair machinery, prolonged G4 stabilization need not compromise nuclear genome integrity, but it can impose persistent transcriptomic shifts and, by stalling mitochondrial replication, deplete mtDNA and trigger mitophagy.

Post-mitotic neurons are uniquely vulnerable. The G4 stabilizer pyridostatin folds a G4 in the Brca1 promoter and downregulates BRCA1 while also repressing autophagy genes such as Atg7; the resulting double-strand breaks cause neurite retraction, synaptic loss, and, in mice, memory deficits and accelerated brain aging (Moruno-Manchon et al., 2020). This toxicity is cell-type-specific, occurring through transcriptional repression in neurons but a replication-dependent route in dividing glia, and it is compounded by the natural accumulation of G4-DNA at oxidized loci in the aging brain, precisely the population most likely to receive these therapies. For synthetic aptamers, off-target binding to unintended receptors and TLR9-mediated inflammation from unmethylated CpG motifs are additional long-term safety concerns for chronic CNS use. Improving selectivity is therefore the central challenge, motivating end-selective and base-surrogate ligands, strand-invading oligonucleotides, and CRISPR-based platforms that direct ligands to individual loci. A cautionary clinical lesson comes from the G4 stabilizer CX-5461, whose principal target at therapeutic doses is in fact topoisomerase II-beta, an off-target interaction associated with the class-wide risks of therapy-related leukemia and cardiotoxicity; such delayed, systemic toxicities make exhaustive, cell-type-resolved safety profiling an essential prerequisite to clinical translation.

### 4.2 From selection to optimization: systematic design principles

A key evolution in the aptamer field is the shift from “selection-only” approaches to selection followed by rational optimization. Systematic datasets analyzing DNA aptamer binding thermodynamics and specificity including collections of hundreds of small-molecule-binding DNA aptamers are providing generalizable insights about what drives high specificity and what trade-offs appear during optimization. Understanding structure–function relationships at this systematic level is essential if DNA is to become a broadly deployable platform rather than a collection of individual success stories. Computational prediction of aptamer structures and binding modes, combined with high-throughput screening and machine learning approaches, promises to accelerate the design cycle and improve success rates.

Increasingly, this optimization is computational rather than purely empirical. In silico secondary- and tertiary-structure prediction, molecular docking, and molecular-dynamics simulation now forecast aptamer folds, receptor-binding modes, and susceptibility to nuclease cleavage, allowing candidate sequences to be triaged before synthesis. Machine-learning models trained on high-throughput binding datasets predict affinity, specificity, and off-target profiles, while high-throughput and organ-on-chip SELEX variants (including BBB microphysiological-system selections) generate the labeled data these models require. Coupling in silico design with rational truncation and chemical modification shortens the discovery cycle and improves candidate fidelity for CNS targets, where the higher bar for brain penetration makes efficient prioritization especially valuable.

### 4.3 Integration with emerging neurotechnologies

The future of molecular origami in neuroscience lies in integration with complementary technologies. DNA aptamers and nanostructures can be combined with optogenetic and chemogenetic tools to create hybrid systems where light or small molecules trigger conformational changes in DNA devices that in turn modulate neuronal activity. Integration with implantable neural interfaces could enable closed-loop systems where electrical recordings inform molecular interventions and vice versa. DNA-based biosensors could provide real-time monitoring of neurochemical states to guide therapeutic interventions, moving toward precision medicine approaches where treatment is dynamically adjusted based on individual patient biomarkers.

Gene therapy vectors, particularly adeno-associated viruses (AAVs), have achieved clinical success for neurological disorders including spinal muscular atrophy (Zolgensma/onasemnogene abeparvovec) and ALS associated with SOD1 mutations (Qalsody/tofersen). DNA origami could potentially serve as non-viral gene delivery vehicles, offering advantages in cargo capacity, targeting flexibility, and reduced immunogenicity compared to viral vectors. Early work on gene-encoding DNA origami for mammalian cell expression demonstrates feasibility, though significant optimization for CNS applications remains necessary.

The gut-brain axis represents another frontier where DNA aptamers could play important roles. Aptamer-based sensors deployed in the gastrointestinal tract could monitor microbiota metabolites, inflammatory markers, or other signals relevant to neurological health. Given emerging evidence linking gut dysbiosis to Parkinson’s disease, Alzheimer’s disease, and other neurodegenerative conditions, the ability to monitor and potentially modulate gut-brain communication at the molecular level could open new therapeutic avenues. DNA aptamers’ programmability and low cost make them attractive candidates for such distributed sensing applications.

### 4.4 Regulatory pathways and clinical translation

The successful clinical translation of DNA-based therapeutics requires navigating complex regulatory landscapes. Macugen (pegaptanib), an RNA aptamer for age-related macular degeneration, demonstrated that aptamer therapeutics can achieve regulatory approval and clinical success, though its subsequent market displacement by anti-VEGF antibodies highlights the competitive pressure from established protein therapeutic platforms. For neurological applications, the higher bar for CNS penetration and the complexity of demonstrating target engagement in brain tissue present additional challenges. Biomarker development, including imaging modalities to visualize aptamer distribution and target binding *in vivo*, will be crucial for advancing clinical programs. As the field matures, establishing standardized manufacturing processes, quality control metrics, and pharmacokinetic/pharmacodynamic models specific to DNA therapeutics will accelerate regulatory pathways.

It is instructive to position DNA-based therapeutics against the two dominant modalities they must compete with. Relative to monoclonal antibodies, DNA aptamers and nanostructures are far smaller, non-immunogenic in most contexts, chemically synthesized with batch-to-batch consistency, thermostable, and natively programmable for reversible deactivation through complementary “antidote” strands, a safety feature with obvious appeal when modulating excitable neural tissue. Relative to small-molecule drugs, they offer higher target selectivity and a rational sequence-to-function design logic, but they lack the passive lipophilic BBB permeability of small molecules and exhibit shorter circulating half-lives, so they depend on the delivery and stabilization strategies discussed above. In practice these platforms are therefore likely to be complementary to, rather than outright replacements for, established protein- and small-molecule therapeutics, with their niche defined by targets that are undruggable by small molecules yet require CNS access that antibodies achieve only inefficiently.

Although agents that directly engage noncanonical DNA structures in the brain remain preclinical, structure-targeted therapeutics have begun to enter human testing in oncology, providing a concrete regulatory template. The G4 stabilizer CX-5461 advanced through a multicenter phase I trial (CCTG IND.231) in patients with advanced solid tumors enriched for DNA-repair deficiencies, establishing a recommended phase II dose, identifying cutaneous phototoxicity as the dose-limiting toxicity, and confirming a synthetic-lethal mechanism through homologous-recombination-dependent responses (Hilton et al., 2022). Such trials, together with the off-target liabilities noted above, define both the promise and the caution required for translating structure-targeted strategies toward neurological indications.

## 5 Conclusions and outlook

The field of molecular origami in neuroscience is transitioning from fundamental discovery to translational application. Naturally occurring noncanonical DNA structures like Z-DNA, G-quadruplexes, and cruciform are now recognized as functional regulatory elements that couple mechanical forces, chromatin states, and transcriptional programs in neurons. Their dysregulation contributes to neurodegeneration through multiple mechanisms: transcriptional disruption, genome instability, aberrant innate immune activation, and sequestration of essential RNA-binding proteins. Understanding these structures as dynamic, context-dependent features rather than static curiosities has revealed new therapeutic targets and mechanistic insights into diseases ranging from Alzheimer’s disease to repeat expansion disorders.

Synthetic DNA aptamers and DNA nanotechnology leverage the same principles of programmable nucleic acid folding to create designer molecules with therapeutic potential. DNA aptamers targeting *α*-synuclein, amyloid-*β*, tau, and key neurotransmitter receptors demonstrate that nucleic acid ligands can modulate disease-driving proteins and neural circuits. DNA nanochannels that gate ion flux in response to external stimuli illustrate how DNA can function as an active neuromodulation device. The ability to design reversible, cell-type-specific, and stimulus-responsive systems using a common chemical platform—one that can integrate recognition, actuation, and sensing functions—represents a paradigm shift from small-molecule and protein therapeutics.

However, significant challenges remain. Achieving robust stability in biological fluids, efficient BBB penetration, cell-type-specific delivery, and appropriate intracellular trafficking requires continued innovation in chemical modification strategies, delivery vehicle design, and targeting mechanisms. Immunogenicity must be carefully managed for chronic neurological applications. Manufacturing scalability and regulatory pathways must be established. Perhaps most importantly, the field must develop systematic structure–function relationships and design rules that enable predictable engineering of aptamer and DNA nanostructure properties rather than relying on trial and error.

The convergence of structural neurobiology, synthetic biology, and nanotechnology creates unprecedented opportunities. As we develop better tools for detecting and manipulating noncanonical DNA structures in living neurons, we gain both mechanistic insights into fundamental neurobiology and new therapeutic handles for intervention. As aptamer selection and DNA nanostructure design become more sophisticated and predictable, we move toward a future where nucleic acid therapeutics are routine components of the neurological pharmacopeia. Integration with complementary technologies—optogenetics, chemogenetics, neural interfaces, gene therapy, and gut-brain axis modulation promises combinatorial approaches that leverage the unique advantages of each modality.

Ultimately, molecular origami approaches offer a unifying framework for understanding how information is encoded not only in DNA sequence but in DNA shape, how this shape can be harnessed for cellular regulation, and how we can engineer nucleic acids to detect, report on, and therapeutically modulate neurological disease. As the field matures from proof-of-concept demonstrations to systematic optimization and clinical translation, molecular origami is poised to become an essential component of precision neuromedicine—enabling interventions that are programmable, reversible, and precisely targeted to specific molecular, cellular, and circuit-level pathologies in the nervous system.

Several genuine controversies temper this outlook and deserve explicit acknowledgment. The physiological prevalence of i-motifs remains debated: antibody-based mapping detects them widely across the genome, yet in-cell NMR reports that i-motif populations are strongly depleted at neutral intracellular pH, so their steady-state abundance in living neurons is unresolved. Similarly, whether the G-quadruplex accumulation seen in Alzheimer’s neurons is a driver of, or merely a consequence of, chromatin decompaction and transcriptional failure is not settled, and Z-DNA has been reported to act as both a transcriptional activator and a repressor depending on genomic context and bound partners. Most consequentially, pharmacological stabilization of these structures is double-edged, producing synthetic-lethal benefit in repair-deficient tumor cells but genotoxic, pro-aging, and neuroinflammatory harm in post-mitotic neurons, where structure-triggered release of cytosolic and mitochondrial DNA can activate cGAS-STING and ZBP1-RIPK1 signaling. These tensions are not yet reconciled and should be read as active questions rather than settled facts.

Progress therefore hinges on a defined set of research priorities and open knowledge gaps: (i) non-perturbing, *in vivo* imaging of noncanonical structures to establish their true dynamics in living brain tissue and resolve the prevalence controversies above; (ii) systematic, cell-type-resolved mapping of the protein complexes that read each structure, to explain context-dependent activator-versus-repressor behavior; (iii) exhaustive long-term safety profiling of G4-stabilizing compounds and DNA nanostructures for genomic stability, mitochondrial and cerebrovascular toxicity, and microglial activation; (iv) truly locus-selective targeting (end-selective ligands, strand invaders, CRISPR-guided delivery) to separate therapeutic effect from genome-wide off-target perturbation; and (v) biocompatible, receptor-mediated BBB delivery vehicles that preserve structural cargo integrity. Addressing these gaps is the prerequisite for molecular-origami approaches to mature from proof-of-concept into precision neuromedicine.

## Glossary

AD: Alzheimer’s disease
ADAR1: adenosine deaminase acting on RNA 1
ALS: amyotrophic lateral sclerosis
ATRX: alpha-thalassemia/mental retardation X-linked
BBB: blood–brain barrier
BG4: G-quadruplex-specific antibody
CD: circular dichroism
ChIP-seq: chromatin immunoprecipitation sequencing
CNS: central nervous system
CUT&Tag: cleavage under targets and tagmentation
DDR: DNA damage response
DHX36: DEAH-box helicase 36
DSB: DNA double-strand break
FMRP: fragile X mental retardation protein
FTD: frontotemporal dementia
G4: G-quadruplex
GBM: glioblastoma multiforme
hnRNP: heterogeneous nuclear ribonucleoprotein
iMab: i-motif-specific antibody
i-motif: intercalated motif
LRP1: low-density lipoprotein receptor-related protein 1
LTP: long-term potentiation
PCBP1: poly(rC)-binding protein 1
PDS: pyridostatin
rG4: RNA G-quadruplex
RMT: receptor-mediated transcytosis
R-loop: RNA–DNA hybrid
SELEX: Systematic Evolution of Ligands by Exponential Enrichment
smFRET: single-molecule Förster resonance energy transfer
TERT: telomerase reverse transcriptase
TfR: transferrin receptor
TLR9: toll-like receptor 9
TMZ: temozolomide
TOP2B: topoisomerase II-beta
Z-DNA: left-handed DNA
ZBP1: Z-DNA binding protein 1
